# Analysis of informal waste management using system dynamic modelling

**DOI:** 10.1016/j.heliyon.2022.e09993

**Published:** 2022-07-16

**Authors:** Kaveri Kala, Nomesh B. Bolia, Sushil

**Affiliations:** a Department of Mechanical Engineering, Indian Institute of Technology Delhi, Hauz Khas, New Delhi, 110016, India; b Department of Management Studies, Indian Institute of Technology Delhi, Hauz Khas, New Delhi, 110016, India

**Keywords:** Informal Waste Management, Circular Economy, Solid Waste Management, Recycling, System Dynamics, Public Policy

## Abstract

The informal sector is the backbone for sustainable waste management in a high population density country such as India. Moreover, the operations of the value chain of informal waste management provide direct or indirect benefits for the environment and human resource development. Unfortunately this sector has always been regarded as a fraudulent activity that sustains without paying taxes, creates unjust competition, and weakens unions and the regulatory structure of the government. These perceptions often lead India to pursue a policy that intentionally or inadvertently amounts to retributive measures. However, the alarming increase in the rate of waste generation has coerced the governments of several countries to incorporate the indispensable informal sector in their policy initiatives. Accordingly, this paper presents a pioneering system dynamics based model (using STELLA Architect software) to analyse the impact of the recent policies and decision strategies on the effectiveness of the informal waste management sector. The paper explores the case of Delhi, India to illustrate the model and provides valuable insights into the urban waste management process. The results of the model demonstrate that significant economic and environmental benefits can be realized by leveraging the natural strengths of the informal sector. Further, it is shown that efficient implementation of policies related to informal waste management can reduce the recyclable waste in the landfills dumped by municipal corporations or otherwise to zero. Also, waste recycling capacity can be increased from 39 percent to 100 percent by strengthening IRC (informal recycling coefficient, introduced in this paper) in a span of 30 years. This increase will have positive impact on land usage, environment degradation and operation cost used in the formal waste collection.

## 1 Introduction

Solid waste management (SWM) is one of the most immediate and grave problems confronting the world. The criticality of this problem can be judged from the fact that twelve out of seventeen UN sustainable development goals (SDGs) can be directly linked to solid waste (Rodić and Wilson, 2017). The growing urbanisation and population makes this problem even worse in developing and transitional economies such as India (Ahsan et al., 2014). Fortunately, developing countries, especially urban areas, have an environmental brigade in the form of informal workers that saves the country from drowning in its own waste. Exemplified by low cost for technologies and processes, high labour, low standardization in the processes (Wilson et al., 2006), this informal sector helps to achieve the SDGs by strengthening the goals of the circular economy. For instance, it reduces the need to depend on virgin raw materials by aiding recycling, it generates employment for poor people and diverts recyclable waste from the landfills and oceans.

To elaborate further, a major portion of total solid waste is municipal or household waste generated from several human activities. In developing countries such as India, most of the municipal solid waste (MSW) is generated in households followed by commercial set ups and market areas (Miezah et al., 2015). Mismanaged municipal waste can become a breeding ground for diseases and spoils environment (Ray et al., 2005). While this is a critical issue that requires day-to-day attention, government agencies have, given their capacity limitations and the large scale of operations, for the most part, been able to address only the collection of the MSW. Waste processing post collection is an area badly in need of attention and overhaul since a sizeable proportion of the waste collected by them effectively ends up in landfills without any significant processing (Lahiry, 2018). The informal sector, on the other hand, is much better at this and has come to assume responsibility for the overall recyclable solid waste. Thus, the informal sector extends a significant support to the circular economy (CE) whose transversal nature, in turn, helps in achieving many diverse goals such as responsible production and consumption, cleaner water bodies and lesser toxic gases from disposal sites.

However, due to a variety of factors, this ecosystem has considerably weakened. These factors include: i) the propensity of state to completely take over MSWM in a bid to buttress its governance and welfare credentials, ii) sheer lack of capacity of the state to deliver at the required scale on one hand and become aware of, engage or appreciate non-governmental stakeholders effectively on the other, iii) exclusion of the informal sectors in policy making, and iv) the inability of the traditional *kabadivalas* to keep up with times and the scale of activities for an end-to-end solid waste management. As a result of this weakening and the expected concurrent inability of the state (through municipal governance) to implement segregation-at-source, a lot of waste generated in India these days does not find its way into the recycling stream and gets blended with different types of municipal solid waste. In this scenario, to leverage the inherent sustainability and strength of the informal ecosystem, including the *kabadis*, it becomes important to strengthen informal waste management through various interventions in policy making.

While some policies and programmes of the government do recognise its efforts, a solution for one often does not work for the other due to the glaring differences between the working of the formal and informal set ups. This is precisely the reason why the urban centres of India need a well thought comprehensive policy framework that not only recognizes the importance of the informal waste management sector but also analyses the nature of relationships between formal and informal sectors. However, the heterogeneity in the value chain of informal sector makes this task an uphill battle. Therefore, in this article, we set out to explore how the effective implementation (with minor changes) of the present policies that are mainly focussed on formal sector, can also be beneficial for informal sector specifically and solid waste management at large. Moreover, to study the informal management system it is important to understand the scope of their activities. However, in the absence of extensive data, this complex system is not easy to capture in any conventional simulation or optimization based model (Miezah et al., 2015). Accordingly, in this paper we use the system dynamics approach to model the informal waste management sector as well as the policies and benefits associated with it, as developing an appropriate policy framework is crucial to facilitate the necessary change in the waste management system. The system dynamics (SD) models are developed using STELLA Architect software. The rest of this paper is structured as follows: Sections 2 & 3 detail the methodology and tests for model validation, section 4 presents the results and section 5 elaborates the corresponding discussion and section 6 concludes the paper with a summary of key findings and possible future extensions.

## 2 Materials and methods

**Figure 1:**
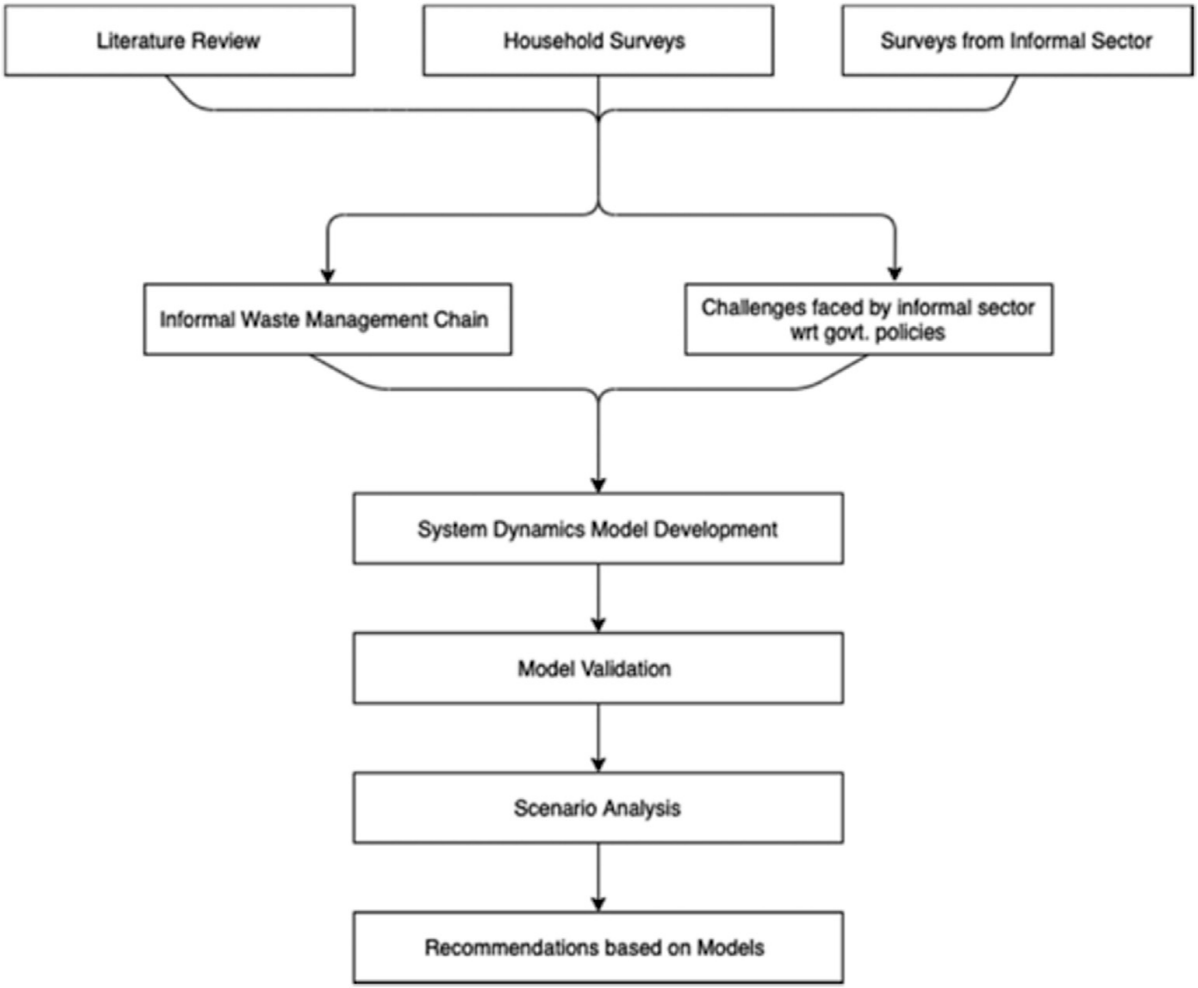
Methodological framework.

As indicated in section 1, in the absence of reliable data especially concerning informal waste management operations, the system dynamics approach is used to evaluate the impact of certain policies on the informal waste management system (IWMS) with a focus on the case of Delhi, India's capital and among its largest urban agglomerations, and the region for which we could collect some basic data used in the SD approach of this paper. The same methodology can be applied to study any other city as well if the relevant data is available. The methodology is presented in Figure 1.

### 2.1 Case background

As mentioned earlier Delhi is the capital city of India. It covers an area of 1484 square kilometers. It is one of the most populous cities in the world with a population size of 30 million and density of 29, 259 people per square kilometer in 2020. According to some official estimates, the amount of municipal solid waste generated is about 10500 metric tons per day (MTD) (IANS, 2019), out of which 83 percent is collected (Singh Sambyal, 2017). Around 29 percent of the collected waste is treated in processing plants and rest is dumped in the landfills. There are three landfills in Delhi, namely, Okhla, Bhalswa and Ghazipur. Like other urban areas of India, Delhi also has two systems for waste collection: formal waste management sector and informal waste management sector. The figures mentioned above account only for the formal sector while there is a significant amount beyond it that is handled by the informal sector (Joshi and Ahmed, 2016). The formal waste management system is driven by five municipal bodies namely, New Delhi Municipal Council, South Delhi Municipal Corporation (SDMC), East Delhi Municipal Corporation (EDMC), North Delhi Municipal Corporation and Delhi Cantonment Board.

More than 50 percent of the waste produced in Delhi is bio-degradable, 20 percent is recyclable and the rest is inert (Kala et al., 2020a, Kala et al., 2020b). All of the biodegradable waste is handled by the formal sector: while the segregated part is transported to composting plants, unsegregated is dumped into the landfills. Most of the recyclable waste, particularly, electronic and plastic is collected and recycled by the informal waste sector (Lahiry, 2019). The larger part of the recyclable waste that is collected by formal sector either goes to waste to energy (WTE) plants or ends up in the landfills.

**Figure 2:**
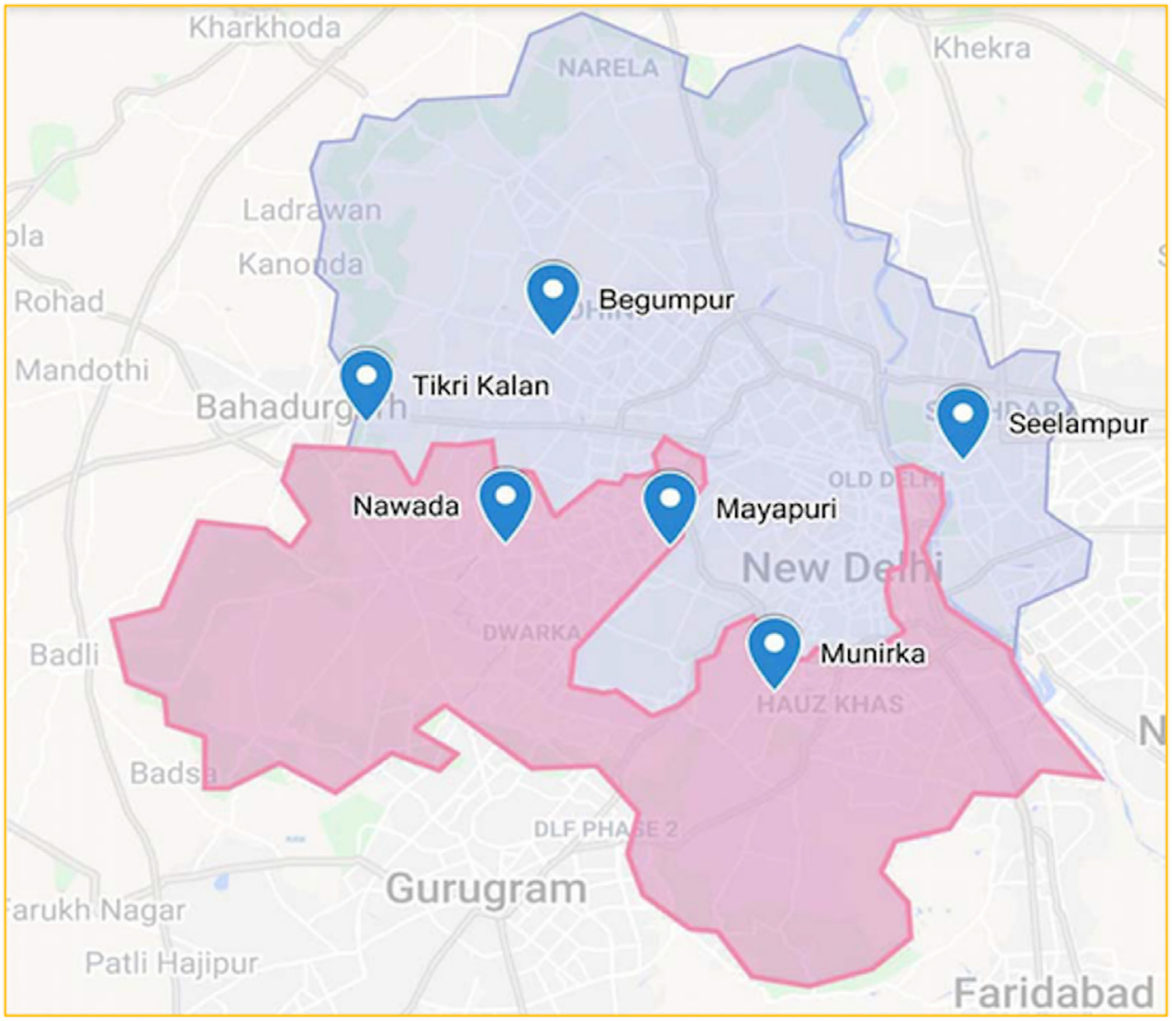
Map of the survey area.

As indicated in the methodological framework, the waste management chain in Delhi is identified with the help of two surveys, namely household survey and survey from the informal sectors. The corresponding areas are highlighted using google my maps (Figure 2). The pink portion highlights the SDMC area considered for the household survey and blue points represent informal setups.

#### 2.1.1 Data collection

*Household Survey*: The first survey is conducted in the households of the socio-economically diverse SDMC area and the other one among the informal waste management actors residing in Delhi. For the household survey, 1000 households are randomly selected, out of which 800 responded. This survey helped in understanding the overall situation of segregation and recycling from the view point of the citizens. Thus, it aided in designing the parameters related to segregation and recycling. The summary of the key results are presented in Table 1.

**Table 1:** Summary of key results from the survey 1.

**Do you segregate waste?**		**Explanation**
Yes	28.6%	Only 28.6 percent of people segregate waste.
No	71.4 %	
***Reasons for not segregating waste?***		
Lack of Time	32.0%	Most of the people are not aware that segregation is not a very involved process and does not require much time or space.
Lack of Space	26.6%	
Lack of Facilities	19.8%	
Expensive Activity	4.3%	
No Incentives	2.6%	
No Reasons	8.1%	
Others	6.6%	
***Incentives preferred for segregation of waste***		
Free Dustbins	38.6%	Overall, among those who currently segregate waste, most belong to the lower or medium income group. However, even among them, segregation can be further increased: these findings reveal that even seeming minor financial incentives would work well for them.
Less Fee	16.6%	
Law mandating segregation	13.6%	
Environmental concerns	12.5%	
Assurance that waste would not be mixed later	11.4%	
Not interested	7.3%	
***Preferred Waste Management Service***		
Recycling	43.9%	Many people do prefer recycling, however only 28.6 percent segregate waste
Composting	36.2%	
RDF	9.8%	
Incineration	5.8%	
Vacuum/Water Sweeping	4.3%	

**Figure 3:**
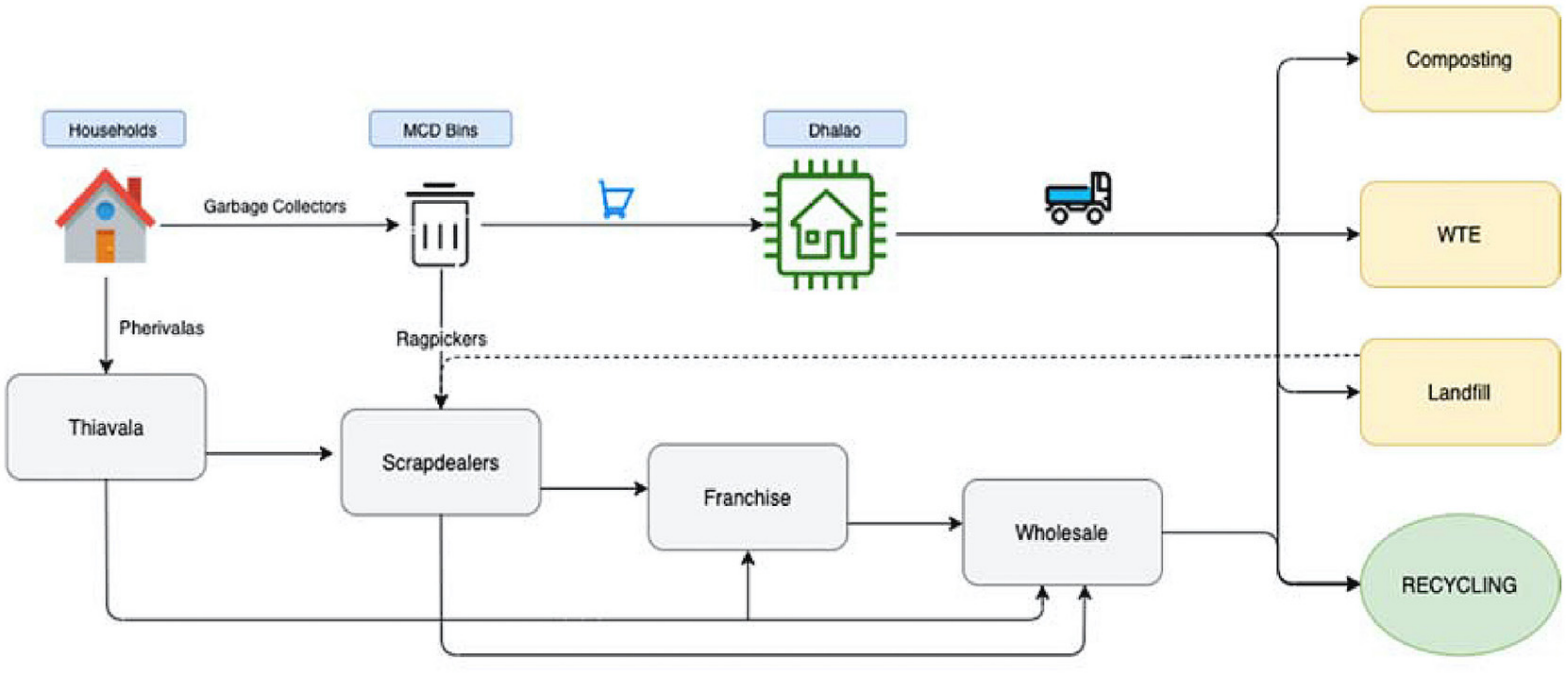
Waste management system in Delhi.

*Informal Sector Survey*: The second survey is conducted among 35 waste dealers in areas (marked as blue points in Figure 2) to incorporate the major hubs of IWMS as well keep the respondent profile diverse. This survey helped in establishing the structure of the waste management chain in Delhi (Figure 3) and identifying the policies affecting the informal waste management. These policies are related to the major challenges faced by the informal sector and further elaborated in section 2.1.2. The survey results are summarized in Table 2.

**Table 2:** Results from the survey 2.

Types of informal actors	Solid waste management rules	Waste to energy scheme	Goods and Services Tax
Ragpickers	0	2	3
Garbage Collectors	1	2	0
*Pheriwala* (Mobile Vendors)	1	4	2
*Thiawala* (Waste Stores)	1	1	3
Scrapdealers (large waste dealers)	1	2	3
Franchise (large waste dealers that mainly deal in contractual assignment)	0	0	2
Wholesalers	1	1	5
Total (%)	14.3	34.3	51.4

Apart from these surveys, a few in-depth interviews are also conducted with officials from SDMC and EDMC. These primary sources combined with secondary sources such as reports and journal publications helped in identifying the course of recyclable material in informal and formal waste management chain in Delhi. Moreover, these surveys helped in estimating the parameters and making better assumptions for our System Dynamics model.

#### 2.1.2 Challenges faced by informal sector

The survey among the waste dealers provides insights on the challenges faced by informal sector. There were questions specifically designed to understand the problems related to the current policy framework. The informal sector primarily faces the following three challenges in carrying out their remarkable role for a financially and environmentally sustainable SWM:

1) Low segregation efficiency: It is observed that even in Delhi, the capital city, the segregation efficiency is quite low. A mere 29 percent of the waste is segregated at source. This statistics is an indicator of low level of awareness among people and that is quite troublesome for the informal waste collector. As waste is not segregated at the source in major parts of Delhi, middlemen and ragpickers segregate the recyclables from mixed waste where they can: at the large dust bins, intermediate waste storage areas and landfills. Consequently, the quantity and quality of reusables and/or recyclables that can be recovered from the waste reduces drastically.
2) High Taxes on recycling and composting: A sharp decrease is observed in the informal revenue generation after the implementation of the GST regime. The survey reveals a significant reduction in the motivation of workers as a result of the introduction of GST on recycled products. On the other hand, several categories of virgin plastic are tax-free, including some that are imported. Thus, in many cases, the cost of the recycled products has become higher than the virgin plastic products. Thus recycling companies, not only have to fight the perception of selling “used/recycled/refurbished, and therefore inferior” products (as before), they are also being forced to compete on price with their virgin product counterparts. Correspon-dingly, buyers of recycled plastics have almost no incentive to go for recyclable, and therefore more sustainable, products. The situation has become bad enough that several recycling and refurbishing units have had to close down.
3) Corporatisation of waste: The municipal corporations of Delhi have given tenders to large private firms to collect and process waste. This conflicts with the interest of informal workers. The WTE plants require a specific amount of waste to function, compelling the private firms to divert huge amounts of waste to these plants. In many instances, this process dislocates rag pickers and other IWMS players, snatches their livelihoods and drives them deeper into poverty. This also happens at the cost of effective segregation, a prerequisite to effective recycling as well as WTE generation, since the corporate players neither have the incentive nor experience to effect segregation, at source, or even later in the waste value chain.

The above-mentioned challenges are connected to the recent policy initiatives namely, Solid Waste Management Rules (SWMR), Goods and Services Tax (GST) and Waste to Energy (WTE) schemes. After carefully evaluating the impact of the policies on the informal waste management sector and brainstorming the possible connections, we have developed the hypothesis presented in section 2.2.

### 2.2 Model development

A causal loop diagram (CLD) exhibits a cause-and-effect relationship among variables. It is a dynamic hypothesis that presents important variables and their relationships (Popli et al., 2017). We have formulated the CLD with the help of information from the literature and primary data collection through surveys. Several logical connections are made to understand the behavior of the real-world system. We have tried to replicate the real informal waste management system through the following hypotheses:

a) Amount of recyclable waste generation is dependent on population and GDP growth.
b) Amount of waste actually recycled (by the informal sector) depends upon the rate of segregation, ease of recycling and motivation amo-ng informal sector (combined as informal recycling coefficient (IRC)).

**Figure 4:**
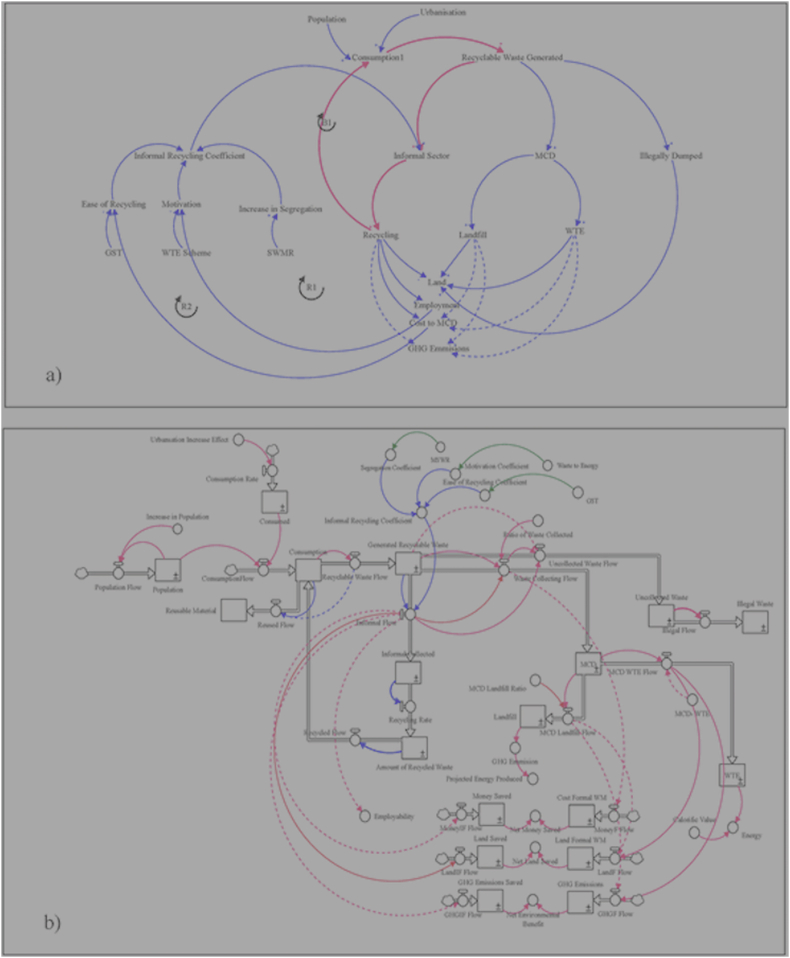
a) Causal Loop Diagram, b) Stock and Flow Diagram.

Effectively, through recycling, the informal sector conserves collective wealth because it avoids the alternative cost of sending and processing/disposing the waste material to a landfill. As highlighted in the literature, we hypothesize that if the recyclable waste goes to the informal sector, it will result in saving land, the environment, and generate employment opportunities (Chintan, 2018). This cost saving brought about the informal sector should be recognized and ploughed back into the IWMS to further facilitate and support the sector. Moreover, increase in employment will motivate people to work. The waste recycled can be consumed again, thus reducing the burden on natural resources. We have incorporated this in our CLD presented in Figure 4 (a). Based on the causal loop formulated, the informal waste scenario of Delhi is modeled using system dynamics. A typical SD model comprises stocks (rectangular blocks), flows (arrows), feedback loops and constraints (Mallick et al., 2014). Figure 4 (b) presents the system dynamics model diagrammatically where as the primary elements involved are explained in Table 3.

**Table 3:** Parameters values for the system dynamics model.

Name and Type	Values (Units)	Formula/Derivation	Source	Explanation
Population (Dynamic) *[pop]*	30,290,936 (people)		(Delhi Population 2020)	The estimates which also represent the urban agglomeration of Delhi (adjacent suburban areas) are taken from the latest revision of UN World Urbanisation Prospects.
Increase in Population (Static) *[inc]*	4.48 % per annum			Deduced by taking population data from 1950 to 2020
Population Flow	per day	$inc*\frac{pop}{365}$	Deduced	
Urbanisation Increase Rate	0.000002739 (per day)		(Das, 2013; WBCSD, 2016; Saraswat and Ghosh, 2017; PTI, 2019; [Bibr bib21][Bibr bib22]; Sannith, 2019)	Rate of increase in consumption of plastic, paper, important metals, textile and glass is estimated per day.
Consumed (kg) *[con]*	0.2 per person (per day)		(Das, 2013; Saraswat and Ghosh, 2017; WBCSD, 2016; [Bibr bib21][Bibr bib22]; Sannith, 2019)	Per capita per day consumption of plastic, paper, important metals, textile and glass is estimated with help of literature available. The metals include mainly steel, copper and aluminium which are a part of our daily lives.
Recycled Flow [rec]	0.95	Amountofrecycled waste	Deduced	Assumption based on surveys from the waste dealers
Consumption Flow	Kg/day	(pop∗con)−rec	Deduced	Static
Recyclable Waste Flow	Kg/day	0.50∗Consumption	Assumed	Static
Motivation Coefficient (Static) *[mot]*	0.35		(Devi et al., 2014)	Motivation was estimated by the quantity of waste collected in a day by the informal sector in Hyderabad assuming similar conditions for Delhi. More than 75kg/day waste is collected by only 3% of the workers 50–75 kg waste is collected by 5% of the workers.25–50 kg waste is collected by 20% of the workers. Less than 25kg/day is collected by 72% of the people. Using these values, it is safe to assume that around 30.875 kgs of average waste is collected by a worker in the informal sector in a day. Assuming that the maximum waste collected by a worker is 75kg/day, the motivation coefficient is estimated to be 0.35.
Ease of Recycling Coefficient (Static) *[*$rec_{coef}$*]*	0.55		(Venkat, 2005)	It was taken little more than the recycling coefficient in US in 1990s.
Segregation Coefficient (Dynamic) *[seg coef]*		0.286	From the field surveys	28.6 percent people segregate in Delhi
Informal Recycling Coefficient *[IRC]*		$\frac{mot_{coef}+rec_{coef}+seg_{coef}}{3}$	Assumed	Waste diverting to the informal sector depends on the three things segregation by citizens, easing the recycling by the government and motivation among the informal sector workers. This assumption has been made after conducting the surveys. The assumed value of IRC give values similar to the current estimates of waste recycled by informal sector.
Informal Flow	Kg/day	$IRC*waste_{rec}$	Deduced	Static
Employability		Informal∗0.075	(Chintan, 2018)	Dynamic
Money saved by informal sector	Rupees	Informal∗5	(Chintan, 2018)	Dynamic
Land saved by Informal sector	Square meter	InformalFlow∗0.000133	(Mudgal, 2015)	Dynamic
GHG Emissions saved (TPD)	TPD	InformalFlow∗0.28	(Sharma and Dikshit, 2016)	Dynamic
Waste Collection Flow		Ratioofwastecollected∗(Recyclablewaste−InformalFlowm2lratio	Deduced	Dynamic
Ratio of Waste Collected	0.83		(Mapping Delhi Landfills, 2020)	Static
m2l Ratio	0.33		Deduced	Static
MCD to Landfill Flow		MCD∗m2lratio	Deduced	Dynamic
m2w Ratio	0.66		Deduced	Static
MCD to WTE Flow		MCD∗m2wratio	Deduced	Dynamic
Energy	Kcal	WTE∗CalorificValue	Deduced	Dynamic
Calorific Value	1400		(GOI, 2017)	Static
GHG Emissions	TPD	m2l∗0.26	(Sharma and Dikshit, 2016)	Dynamic
Projected Energy produced	J	GHGEmissions∗0.045	(Deshpande, 2015)	Dynamic
Uncollected Waste Flow	Kg/day	RecycledWaste−(WasteCollectionFlow+InformalFlow	Deduced	Dynamic
Illegal Flow	Kg/day	UncollectedWaste∗0.99	Deduced	Dynamic

Although the measurement of the waste quantity at the final disposal sites is the commonly used approach, this method neither accounts for the sizable amount of recyclable waste managed by the informal sector before the ultimate disposal, nor the absence of municipal services in slums and rural areas where the waste is thrown away illegally at open-sites. Therefore, the basis of this stock and flow diagram is the amount of recyclables consumed by the people. Figure 4 (b) presents the Stock and Flow Diagram (SFD) for the IWMS in Delhi.

## 3 Model tests and validation

Once a model is developed, it needs to be verified to build a certain level of confidence. To build such confidence, a model's structure and behavior should be in accordance with the real-world structure and behavior of the system (Bala et al., 2017).

### 3.1 Tests for model structures

The following tests are performed for the model structure:

a) Structure Verification Test: This test is an empirical way to compare the relations expressed by the model with those in the real world. For the structural verification of our model, the survey results are utilised, as described in sections 2 and 2.2. Further, our model is evaluated against the previous models and data available in the literature.
b) Parameter Verification Test: This test is performed to estimate the constant parameters such as initial value of the stocks, constant values of the converters and table functions. It utilizes the data available in the literature and compares it with the existing system both numerically and conceptually. Table 3 highlights the sources and logical explanation behind the values of the parameters.
c) Dimensional Consistency Test: To ensure that the measurement units of all the variables and constant used in the model are dimensionally consistent, each equation is tested separately. The right-hand side of the equation should be dimensionally equal to the left-hand side of the equation and also the dimensions of the variables/constants should represent their actual meanings. The measurement units of all the variables are presented in Table 3 to establish the dimensional accuracy of our model.
d) Extreme Condition Test: This test is done to realize if the model works in an extreme condition. In this case, some of the inflow parameters were taken to be zero to understand the behaviour of the model and see if it is in accordance with the expected outcome. In particular, when the value of informal recycling coefficient is kept zero (i.e., motivation coefficient = 0, ease of recycling coefficient = 0 and segregation coefficient = 0), the amount of recyclables diverted towards the informal sector also becomes zero. As expected, under this scenario, the waste goes either to the formal sector or is illegally dumped, the amount of waste recycled with the current infrastructure reduces dramatically and the amount of waste dumped in landfills increases. All of this, as clearly expected, results in the environment and economic benefits associated with the informal sector coming down to zero.

### 3.2 Tests for model behavior

**Figure 5:**
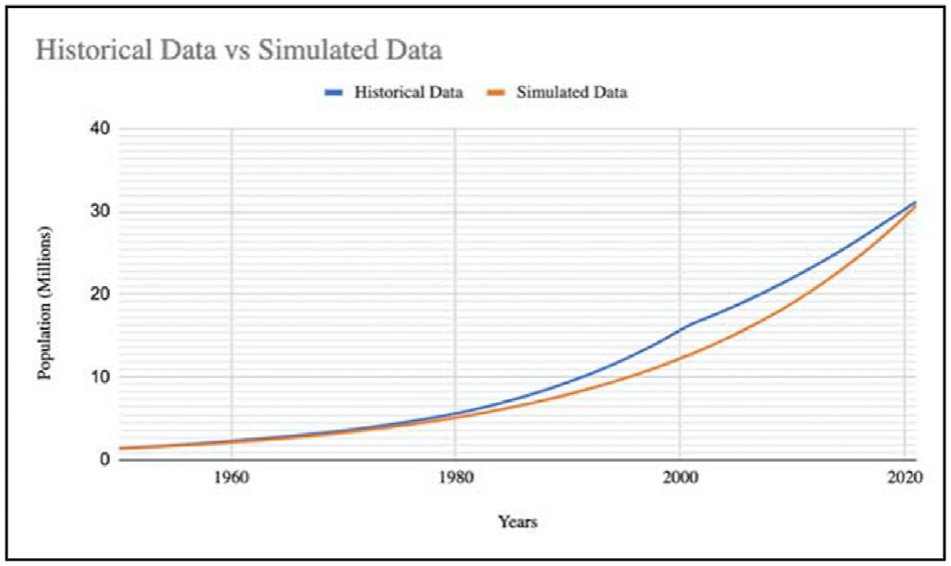
Validation graph.
b) Behavior Anomaly Test: When the model contradicts with the actual situation primarily because of errors in assumptions, this test is performed. These anomalies can be identified by carefully examining the loops. Since our model is able to reproduce the behavior of the system in real-world, it can be safely assumed that it is free from anomalies and the assumptions leading to it are backed up by sound logical reasoning.
a) Behavior Sensitivity Test: Behavior sensitivity test is done to check the robustness of the model. It ascertains whether the plausible changes in the value of the parameters lead to failure in the behavior tests mentioned above. Ideally the system should be insensitive to the changes in parameter values, however, both the real system and the model are sensitive to a few parameters. The parameters leading to change in a model are pivotal in determining the improvement strategies since they had a real impact on model. The primary aspect of our current model is to maximize the diversion of the recyclable waste to informal sector. Thus, all the parameters are tested on the basis of this variable. In this model the parameter values are changed within the range of (+-15%). It is observed that the model results are primarily sensitive to two parameters namely, rate of increase in population and informal recycling coefficient (presented in Figure 6).

Any model that represents an actual operation should not only look but also behave like one. After confirming the structure, the following tests are performed to verify the behavior replication ability of the model:

a) Behavior Reproduction Test: This test compares the model generated behavior with the observed behavior of the real world. The accuracy of this test determines the level of confidence in the model. It primarily focusses on pattern prediction rather than point prediction. The picture of waste management in India has always been confusing because of scanty and scattered data available in public domain, therefore, we have used the data of population as reference. The trend from the population data available in the literature is compared to the trend formed by the simulation. The reliability of this model is justified by the reproduction of the pattern from the historical data as presented in Figure 5. Thus, the model can replicate the real behavior of the system quite accurately. Therefore, our model can be used for policy analysis.

**Figure 6:**
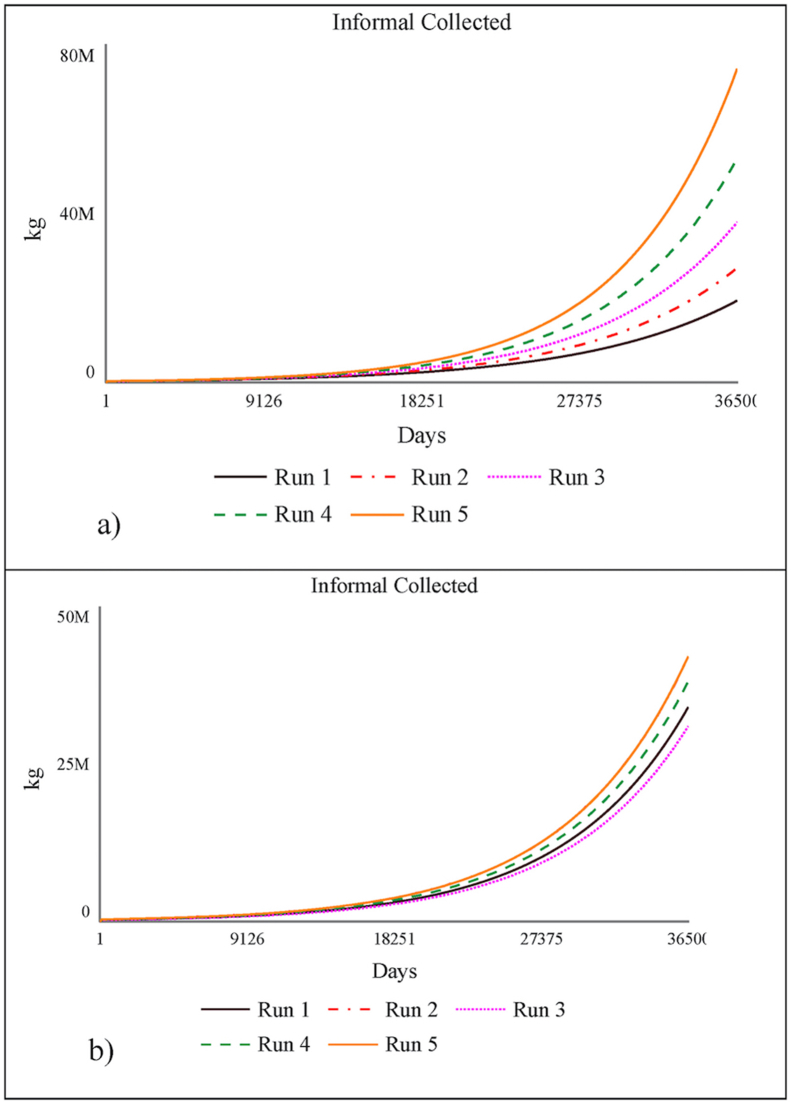
Sensitivity Analysis a) Increase in Population b) Informal Recycling Coefficient.

The following conclusions can be drawn from the above:

a) No significant change is observed by changing the parameter within ±15 range. This means that the model is insensitive to most of the parameters and is thus robust.
b) The parameters that were assumed due to the lack of data have no significant effect on the result, thus justifying the assumptions.
c) The parameters having the maximum effect on the result are deduced from the published literature and primary data collection through surveys.
d) The parameters with the maximum effect (in isolation and combination) are used as the pivotal points for the policy analysis in the next section.

## 4 Results

Based on the analysis of the model validation tests, five scenarios are considered, namely, business as usual (BAU) scenario, SWMR scenario, GST scenario, WTE scenario and comprehensive management policy (CMP) scenario.

1) **BAU**: The current base-line simulation results are able to mimic and explain the actual observations. This scenario is a general continuation of the current circumstances without any additional policy benefits. The current pivotal policy parameters are presented in Table 4.

**Table 4:** Pivotal policy parameters.

Scenario	Ease of Recycling	Motivation Coefficient	Segregation Coefficient	Informal Recycling Coefficient	Delay Time Considered
BAU	0.55	0.35	0.29	0.39	-
SWMR	0.55	0.35	1	0.63	5 years
GST	1	0.35	0.29	0.55	5 years
WTE	0.55	1	0.29	0.61	5 years
CWM	1	1	1	1	5 years
2) **Intervention 1** (Effective implementation of SWMR, 2016): Municipal Solid Waste (Management and Handling) Rules, 2000, were updated after sixteen years in the form of Solid Waste Management Rules (2016). The word “municipal” was removed as the jurisdiction of the rules has been expanded beyond municipal areas. One of the most significant changes in the new solid waste rules is the identification of the duties of the waste generator. Further, segregation has been emphasized as one of the most critical steps towards effective waste management, and urban local bodies have been made responsible to ensure segregation of waste at source (MoEF, 2016). It is well understood that segregation at source is crucial for better efficiency in the processing of waste. All the stakeholders such as citizens and private institutions are required to team up with the respective ULBs and support segregation at source. In addition, bulk generators (MSW>5000MT) need to manage organic waste measures such as composting while the recyclable waste need to be given to *kabadivalas* or authorised recyclers There is also a penalty for non-segregation in the new rules. If these aspects of segregation are properly implemented by the authorities and are also adopted by the citizens, the waste management system will be entirely transformed (Kala and Bolia, 2020). This will also present an impeccable opportunity to link all the stakeholders and benefit from a hybrid (combination of centralised and decentralised) waste management system. The amount of recyclable waste collected and recycled is also dependent on percentage of segregation indicated by segregation coefficient in our model. If SWMR are implemented effectively, then percentage of segregation (ideally 100) will increase which would lead to increase in segregation coefficient and eventually more waste would go to the informal sector. However, given the population density and other complications, it would take some years to implement these rules. Accordingly, a delay of five years is introduced to accommodate for a delay in effective implementation of these rules. The pivotal policy parameters are presented in Table 4.
3) **Intervention 2** (Removal of GST on recycling): GST is a consumption tax (or indirect tax) on the supply of goods and services. The government argues that the introduction of taxes on the recycling and composting industry through the GST regime is meant to address tax evasion. However, the informal chain has had to face significant revenue reduction due to GST (Chintan, 2018). This has been severe enough that several units have had to actually shut down, as already indicated in section 2.1.2. The critical role of the informal sector in waste management, and recycling in particular, is already elaborated in section 1. Recycling itself is crucial for environment and waste management. Further, due to perverse incentives and the structure of this ecosystem, it is far more expensive to recycle used plastic than producing virgin plastic. The expense of what disposal of plastic does to the environment is not accounted for in the expense of virgin plastic while the cost of gathering, sorting, collecting, cleaning and recycling plastic is captured in the cost of recycled material. Under such circumstances it is evident that recycling needs policy support (The Economic Times, 2019). Thus, a GST removal scenario is assumed in which the ease of recycling coefficient is increased from 0.55 to 1, producing an effect of 15% increase in the waste collected by the informal sector. The pivotal policy parameters are presented in Table 4.
4) **Intervention 3** (Effective Implementation of Waste-Energy Scheme): Most of the WTE plants do not comply with the SWMR, 2016 that assert only segregated non-recyclable high-calorific segments be sent to these plants. Moreover, the Solid Waste (Management and Handling) Rules 2016 call for recycling as a key strategy, something clearly not possible if the recyclable waste is extensively used for technologies such as WTE (Ijgosse, 2019). Such corporatization of waste disrupts the whole value chain and leaves less recyclable products for informal sector. This demotivates the informal workers and they start doing other jobs. Though India does need scientific and sophisticated methods to process the huge quantity of waste, it is the decentralised system that will prove to be more efficient in dealing with this problem because of the following reasons:
  a) High organic content: The organic content in the MSW is more than 50% because of the cultural values of consuming less processed and packaged food as compared to cooked food. Though this trend in the food habits might change in future, as of now Indian MSW is soggy. This organic content in the MSW reduces the calorific value by 30 percent as compared to the recyclable waste (Swaminathan, 2018). While calorific value of 2000kJ/kg is required for efficient working of WTE, Indian MSW in the absence of effective segregation will only yield around 1400 kJ/kg.
  b) High cost of construction: To realize the benefits of waste management with respect to environment, the WTE plants are required to be extremely regulated for toxins. Other obstructions in the way of a successful decentralised waste management system are high cost and scale of WTE projects. The pivotal policy parameters are presented in Table 4.
5) **Intervention 4 (Comprehensive waste management):** The last case is the comprehensive management scenario. If all the three interventions are combined effectively, most of the recycled waste can be diverted to the informal sector in a span of 30 years. A comparison among all the scenarios reveals that optimizing any one of the policies will surely decrease the amount of recyclable waste dumped to landfills or vacant plots (illegal) but combining the maximum effect of all the policies will provide significantly better results. Figure 7 and Table 4 presents the comparison among different scenarios.

**Figure 7:**
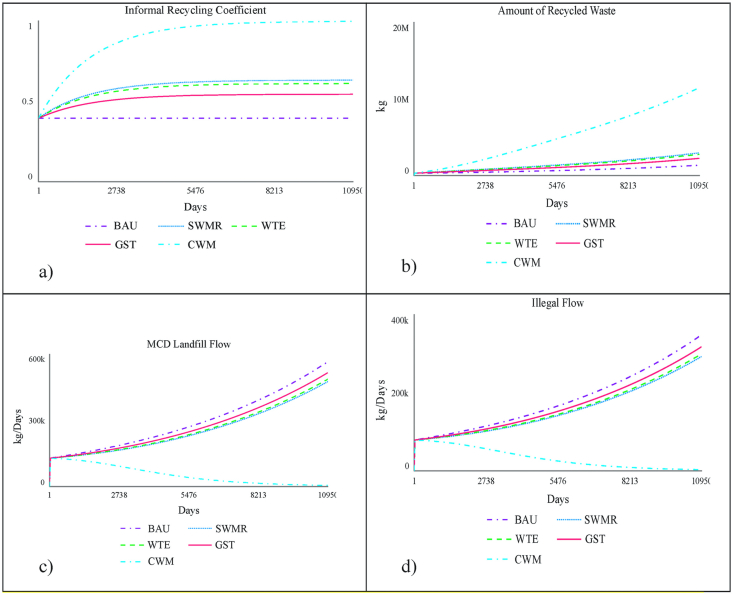
Comparative Intervention Graphs a) Informal Recycling Coefficient, b) Amount of Recycled Waste, c) Landfill Flow of Municipal Waste, d) Illegal Flow.

## 5 Discussions

Thus, it is clear from section 4 that an effective implementation of SWMR, more stringent guidelines for WTE plants and an improved tax regime with respect to recycling can significantly waste management in Delhi. However, to ensure this proper systems are required to be put in place for effective compliance, something that has remained elusive for the government in India. The pivotal policy parameters mentioned in the results section can be improved through the following measures:

**Segregation Coefficient (SC)**: Segregation coefficient is associated with the percentage segregation and is affected by the implementation of SWMR. While the rules are noteworthy for their acknowledgment to integrate informal sector with formal waste management system, there is little guidance on the course of action. There are no bye-laws, or even recommendations, that state how the informal sector can be mainstreamed and integrated with the formal waste management ecosystem. Therefore, detailed bye-laws should be provided by the state governments according to the condition of their cities. Moreover, the municipal corporations should, to the extent possible, work with collectives in the informal sector for arranging door-to-door collection and segregation of household waste and providing material recovery facilities (MRFs) and personal protective equipment (PPE) for secondary segregation. Since citizens also play a major role in source segregation, they should be made aware about the importance of segregation through relevant communication channels (Kala et al., 2020a, Kala et al., 2020b). More stringent penalties should be imposed on households for non-segregation of waste.

**Ease of recycling coefficient (ERC):** This coefficient is directly connected to the tax regime. Clearly, the ERC will tend to decrease due to taxes on recycling activities. To improve this coefficient, taxes that reflect environmental cost should be imposed on virgin plastic and tax rebates provided to recycling activities in recognition of the benefits they bring to the environment. Although a detailed policy prescription can only be based on a detailed analysis beyond the scope of this paper, higher taxes (increased GST rate and/or cess) on virgin plastic, along with a zero or bare minimum GST rate on recycled plastic should be considered. The money collected from taxes imposed on waste management can go towards supporting the informal sector. The recycling policy should also be based on the polluter-pays principle, burdening waste generators to pay for its removal. The industries whose products are difficult to reuse or recycle should be levied higher tax (Walani, 2017). Moreover, recycling constitutes just one but important part of the loop of the circular economy. Clearly, the more the transactions in this part, the higher is the sustainability of the economy. However, the demand for recycled material competes with that of virgin materials, where the latter has an unfair upper hand. Therefore, to successfully apply principles of circular economy and gain from its sustainability benefits, India needs to evolve appropriate structures for the recycling ecosystem, particularly with regard to its taxation, corresponding adaptation of the GST framework, and including a complete exemption where needed and possible.

**Motivation Coefficient (MC):** This coefficient is dependent, among other factors, on the job opportunities and salary of the informal workers. Thus, this coefficient is associated with corporatisation of waste for WTE plants. The corporatisation of waste processing may seem to be administratively convenient on the face of it, but is hardly sustainable (explained in section 3). For one, it leads to the informal sector losing access to waste material and, of course, losing employment opportunities. Complete corporatisation could also financially be less rewarding: while the informal sector may actually pay for access to the waste, corporate responses to tenders floated by ULBs often seek remuneration for collecting waste. Further, given the decentralized nature of its operations, and the financial incentive it offers, which the corporate collection does not, the informal sector is better placed than the corporate sector to effect behavioural changes associated with segregation at source. While the concept of generating energy from waste seems to be a quick fix solution, the government should empower the, locally designed, culturally apt and cost-effective solutions through research and development.

**Figure 8:**
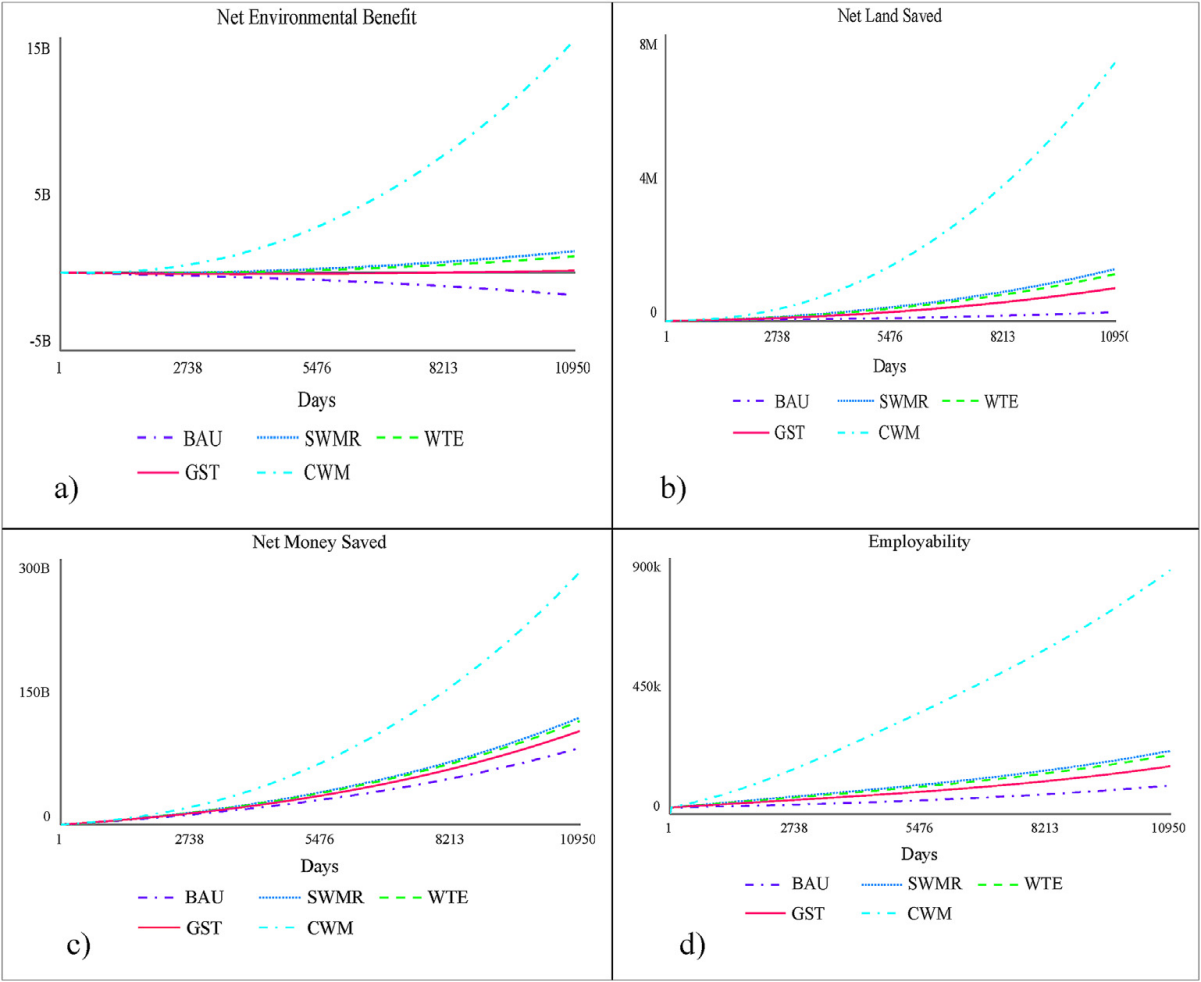
Comparative Graphs a) Net Environmental Benefit, b) Net Land Saved, c) Net Money Saved, d) Employability.

It is already known that actors of the informal value chain recognised and supported by government agencies and organised into strong cooperatives, appear to have higher incomes than other informal workers. In fact, many cities such as Pune have done remarkably well in this context. The SWaCH model helps Pune Municipal Corporation (PMC) in door-to-door collection and segregation while integrating informal waste workers (PMC, 2016). This model is not only cost effective but also recovers resources in a sustainable manner. Thus, government support to informal waste management system can help realize significant environmental and economic benefits (logistic and land costs). Figure 8 compares the benefits provided by the informal sectors under the ideal values of pivotal policy parameters.

## 6 Conclusion

The informal sector is extremely important to manage waste, particularly in a high population density country such as India. Any country with a similar population size and infrastructural facilities can be crushed under the weight of its own waste without the support of the informal sector. Due to their unrecognized efforts, the informal sector players have managed to delay the externalities of poor waste management. However, in the past few years waste has increased at an unsustainable rate while the support to informal sector has remained the same if not decreased. The mounds of waste at the landfills clearly call for fundamental changes in the waste management system.

Many existing environmental policies and laws acknowledge waste management directly or indirectly. However, these policies in their current form are not comprehensive enough since they tend to ignore the informal sector almost completely. The few that do acknowledge the informal sector are poorly implemented and often weaken the informal sector instead of strengthening it. These policy instruments predominantly fall short in terms of defining mandatory rules or precise descriptions, the specific measures to be implemented and the targets to be achieved. Clearly, India needs fundamental changes in its current policy and regulations framework that leverage its potentially strong informal sector. Through this paper, we have identified the recent policies, and pivotal factors associated with them, that have a potential to improve the condition of informal waste management in Delhi. The System Dynamics model of the paper is validated and clearly demonstrates that strong performance improvement is possible by strengthening and leveraging the promise of the informal sector.

Future extension of this work includes a more elaborate integration model using system dynamics that can analyse the potential of informal and formal sector working together to expand recycling capabilities for achieving goals of circular economy.

## Declarations

### Author contribution statement

Kaveri Kala: Conceived and designed the experiments; Performed the experiments; Analyzed and interpreted the data; Wrote the paper.

Nomesh B. Bolia, Sushil: Contributed reagents, materials, analysis tools or data.

### Funding statement

This research did not receive any specific grant from funding agencies in the public, commercial, or not-for-profit sectors.

### Data availability statement

Data will be made available on request.

### Declaration of interests statement

The authors declare no conflict of interest.

### Additional information

No additional information is available for this paper.
